# H_2_O_2_/O_2_ self-supply and Ca^2+^ overloading MOF-based nanoplatform for cascade-amplified chemodynamic and photodynamic therapy

**DOI:** 10.3389/fbioe.2023.1196839

**Published:** 2023-05-24

**Authors:** Yujia Liang, Zhengmin Cai, Yamei Tang, Chenglin Su, Liye Xie, Yan Li, Xinqiang Liang

**Affiliations:** Guangxi Medical University Cancer Hospital, Nanning, China

**Keywords:** CaO_2_, MOFs, H_2_O_2_/O_2_ self-supply, chemodynamic therapy, photodynamic therapy

## Abstract

**Introduction:** Reactive oxygen species (ROS)-mediated therapies have typically been considered as noninvasive tumor treatments owing to their high selectivity and efficiency. However, the harsh tumor microenvironment severely impairs their efficiency.

**Methods:** Herein, the biodegradable Cu-doped zeolitic imidazolate framework-8 (ZIF-8) was synthesized for loading photosensitizer Chlorin e6 (Ce6) and CaO_2_ nanoparticles, followed by surface decoration by hyaluronic acid (HA), obtaining HA/CaO_2_-Ce6@Cu-ZIF nano platform.

**Results and Discussion:** Once HA/CaO_2_-Ce6@Cu-ZIF targets tumor sites, the degradation of Ce6 and CaO_2_ release from the HA/CaO_2_-Ce6@Cu-ZIF in response to the acid environment, while the Cu^2+^ active sites on Cu-ZIF are exposed. The released CaO_2_ decompose to generate hydrogen peroxide (H_2_O_2_) and oxygen (O_2_), which alleviate the insufficiency of intracellular H_2_O_2_ and hypoxia in tumor microenvironment (TME), effectively enhancing the production of hydroxyl radical (•OH) and singlet oxygen (^1^O_2_) in Cu^2+^-mediated chemodynamic therapy (CDT) and Ce6-induced photodynamic therapy (PDT), respectively. Importantly, Ca^2+^ originating from CaO_2_ could further enhance oxidative stress and result in mitochondrial dysfunction induced by Ca^2+^ overloading.

**Conclusion:** Thus, the H_2_O_2_/O_2_ self-supplying and Ca^2+^ overloading ZIF-based nanoplatform for cascade-amplified CDT/PDT synergistic strategy is promising for highly efficient anticancer therapy.

## 1 Introduction

Cancer is one of the most lethal diseases and causes millions of deaths annually with increasing mortality worldwide. Considering the high risk and death rate of cancer, scientists around the world have dedicated themselves to achieving effective and precise diagnoses as well as safe and hazard-free therapy to fight against it. With the rapid development in nanotechnology over the past 2 decades, nanomaterials have provided an advanced approach from anti-cancer experts and are expected to be used in cancer imaging and treatment therapy. (Wang et al., 2021a; Yang et al., 2021a; Zhang et al., 2021; Zhou et al., 2021; Shan et al., 2022; Zhang et al., 2022; Li et al., 2023; Lu et al., 2023). Metal-organic frameworks (MOFs) with potential biological performance, such as biocompatibility, cytotoxicity, and biodistribution, have been extensively studied in nanotherapeutics. (Wang et al., 2019a; Xie et al., 2019a; Yang et al., 2019a; Zhang et al., 2019a; Zhao et al., 2019; Pandey et al., 2020). MOFs are a series of crystallized porous materials coordinated by metal-containing cores (e.g., metal ions and clusters) and organic linkers (e.g., carboxylate ligands, phosphonates, sulfonates, and other negatively charged ligands). MOFs are not only good carriers of nanocargo (drugs and contrast agents) because of their porous and oriented structure but also contrast agents themselves due to their multifunctional building blocks. (Wang et al., 2019b; Cai et al., 2019; Ren et al., 2019; Rojas et al., 2019). Importantly, with good biodegradability and biocompatibility, MOF composites could be constructed as physiological environment-accommodative synergist therapy platforms. (Wang et al., 2021b; Liang et al., 2021; Bian et al., 2022). Because of this, incorporating functionalized compositions and comprehensive structures within MOFs to obtain nanoplatforms with collective properties and advanced performance has attracted much attention.

As a major molecule produced during oxidative stress, reactive oxygen species (ROS) contains singlet oxygen (^1^O_2_), superoxide anions (O_2_^−^), and hydroxyl radicals (•OH), which are considered to be essential factors in the occurrence, development, and recurrence of cancer. (Yang et al., 2019b; Li et al., 2021a; Tao et al., 2022; Truong Hoang et al., 2022; Yu et al., 2022; Cao et al., 2023). Furthermore, depending on their high selectivity and unrecognized drug resistance, ROS-mediated therapies such as chemodynamic therapy (CDT) (Zhao et al., 2021a; Yang et al., 2021b; Zhou et al., 2021) and photodynamic therapy (PDT) (Zhang et al., 2019b; Zhao et al., 2021b; Rui et al., 2021) have been considered as noninvasive anti-cancer treatments. CDT utilizes the Fenton/Fenton-like reaction between catalysts and hydrogen peroxide (H_2_O_2_) to generate cytotoxic •OH, (Chen et al., 2020; Wang et al., 2020; Cao et al., 2021), while PDT relies on nontoxic photosensitizers that are activated by visible or/and near-infrared (NIR) light to convert oxygen (O_2_) to ^1^O_2_. (Deng et al., 2017; Xie et al., 2019a; Yang et al., 2019c; Monro et al., 2019; Sivasubramanian et al., 2019). However, the harsh tumor microenvironment (TME) is an obstacle against achieving highly efficient therapeutic efficacy. Compared to normal cells, TME exhibits unique characteristics, such as mildly acidic conditions (pH = 5.5–6.5), internal hypoxic environment, high levels of intracellular glutathione (GSH, ∼10 × 10^−3^ M), excessive H_2_O_2_, (50−100 × 10^−6^ M), and hypoxia conditions. (Wang et al., 2018; Peng et al., 2021; Chang et al., 2022). The low intracellular H_2_O_2_ concentration and inherent hypoxia at tumor sites result in the low ROS production efficiency of CDT and PDT, respectively. In addition, the strong antioxidant GSH in TME also would downregulate the ROS level, aggravating the attenuation of antitumor efficiency. Li et al. loaded the chemotherapy prodrug disulfiram (DSF) and coated glucose oxidase (GOD) on the surface of Cu/ZIF-8 nanospheres and finally encapsulated manganese dioxide (MnO_2_) nanoshells to achieve efficient DSF-based cancer chemotherapy and dual-enhanced CDT. The MnO_2_ layer could achieve GSH depletion and relieve tumor hypoxia in the TME, the released Mn^2+^ could initiate *T*_1_-MRI for the tracking of the nanocatalyst *in vivo*, and the O_2_ produced in the reaction could oxidize glucose to H_2_O_2_ and gluconic acid in the presence of GOD. (Li et al., 2021b). Thus, engineering H_2_O_2_/O_2_ self-supplying therapeutic nanoplatforms to increase *in situ* the H_2_O_2_ and O_2_ concentration at tumor sites and constructing a CDT/PDT strategy to achieve a more synergistic effect than that of single-mode might be possible solutions.

More attractively, most of the latest research has provided approaches to improve the propagation of H_2_O_2_ and relieve hypoxia at tumor sites. (Wang et al., 2019c; Liu et al., 2019). Among them, a highly biocompatible metal peroxide, calcium peroxide (CaO_2_), has received widespread attention because of its excellent advantages, such as the simultaneous generation of O_2_ and H_2_O_2_ immediately following a reaction with water, serving as a donor of H_2_O_2,_ and eliminating GSH in response to TME. (Sun et al., 2021a; Sun et al., 2021b; Liu et al., 2022a; Liu et al., 2022b). Additionally, overloaded exogenous Ca^2+^ could induce mitochondrial damage and further disorder the oxidative stress, resulting in the imbalance of calcium transport channel and accelerating tumor calcification-mediated apoptosis. (Zhang et al., 2019c; He et al., 2021; Wan et al., 2021; Docampo and Vercesi, 2022; Zheng et al., 2022). Hence, CaO_2_ could be appreciated as an advanced candidate for the rational design of multifunctional nanoplatforms for promoting CDT and PDT efficiency while achieving mitochondrial-localized Ca^2+^ overloading, ultimately allowing amplification of intracellular ROS-mediated therapeutic effect. (Hu et al., 2020; Shen et al., 2021; Chen et al., 2022).

Zeolitic imidazolate framework-8 (ZIF-8), composed of the coordination of Zn ions with 2-methylimidazole (2-MeIM), is a promising MOF for the construction of therapeutic nanoplatforms. (Xie et al., 2019b; Qin et al., 2019; Yang et al., 2020; Wang et al., 2021c; Jiang et al., 2022; Li et al., 2022). In this study, the biodegradable Cu-doped ZIF-8 was synthesized for loading photosensitizer Chlorin e6 (Ce6) and CaO_2_ nanoparticles (NPs), followed by surface modification by hyaluronic acid (HA), finally obtaining HA/CaO_2_-Ce6@Cu-ZIF nano platform. Once HA/CaO_2_-Ce6@Cu-ZIF targets tumor sites through HA-mediated active endocytosis and degrading by hyaluronidase (HAase), the degradation of Ce6 and CaO_2_ is released from the HA/CaO_2_-Ce6@Cu-ZIF in response to the acid environment, while the Cu^2+^ active sites on Cu-ZIF are exposed. The released CaO_2_ decompose to generate H_2_O_2_ and O_2_, which alleviates the insufficiency of intracellular H_2_O_2_ and hypoxia in TME, effectively amplifying the production of •OH and ^1^O_2_ in Cu^2+^-mediated CDT and Ce6-induced PDT, respectively. Importantly, Ca^2+^ originating from CaO_2_ could further amplify the oxidative stress and lead to mitochondrial dysfunction induced by Ca^2+^ overloading. Thus, the H_2_O_2_/O_2_ self-supplying and Ca^2+^ overloading MOF-based nanoplatform for cascade-amplified CDT/PDT synergistic strategy is promising for highly efficient anticancer therapy.

## 2 Experimental section

### 2.1 Chemicals

Zn(NO_3_)_2_•6H_2_O (0.1 M), 2-MeIM (C_4_H_6_N_2_, 99%), Cu(NO_3_)_2_•3H_2_O (AR), CaCl_2_ (97%), and HA (10 k) were purchased from Shanghai Aladdin Technology Co., Ltd. Ce6, DAPI, MTT, calcein-AM, and PI were supplied by Sigma-Aldrich. The annexin V-FITC/PI apoptosis kit was obtained from MultiScience Biotech Co., Ltd. All liquid chemical reagents were used without further purification.

### 2.2 Synthesis of CaO_2_ NPs

CaO_2_ NPs were obtained by a hydrolysis–precipitation process. A specific amount of CaCl_2_ (1 g) was sent into the HA (50 mL, 0.1 M) solution at room temperature under continuous stirring for 30 min. After that, NH_3_•H_2_O (5 mL, 1 M) and H_2_O_2_ (1.5 mL, 30%) were sequentially injected and synthesized for 3 h. Afterward, NaOH (1.0 mL, 1 M) was added under ultrasound. Finally, the CaO_2_ NPs were purified by centrifugation (13,000 rpm, 10 min) and sequentially washed with NaOH solution, pure water, and anhydrous ethanol three times.

### 2.3 Synthesis of HA/CaO_2_-Ce6@Cu-ZIF

The HA/CaO_2_-Ce6@Cu-ZIF was prepared via an unfussy one-step method. A specific Zn(NO_3_)_2_•6H_2_O (300 mg) and Cu(NO_3_)_2_•3H_2_O (50 mg) were dissolved in methanol (100 mL) and formed an uniform solution. Then, 2-MeIM (190 mg), HA-stabilized CaO_2_ NPs (50 mg), and Ce6 (20 mg) dissolved in the methanol solution (100 mL) were added drip by drip and reacted for at least 30 min under N_2_ atmosphere. Finally, the HA/CaO_2_-Ce6@Cu-ZIF was collected by centrifugation (13,000 rpm, 10 min) and washed with methanol three times.

### 2.4 Characterizations

TME images and corresponding elemental mapping were collected from Tecnai T20 at an accelerating voltage of 200 kV. The size of nanoparticles was calculated using Image J for 100 counting number. XRD patterns were obtained from Bruker D8 ADVANCE (Cu Kα radiation (*λ* = 0.154 nm) at 40 kV and 40 mA. Zeta potential and DLS measurements were gained by Zetasizer Ultra with He-Ne laser (633 nm). UV-vis absorption spectra were acquired from Shimadzu UV-1601. XPS spectra were analyzed from Rigaku DMAX-2400. FT-IR spectrum was accumulated from Nicolet Avatar 360 with the KBr wafer technique. ICP-OES measurements were surveyed from iCAP 6000 series. CLSM images were captured from Leica SP8. Flow cytometry was measured using BD accuri C6.

### 2.5 ROS generation estimation

The generation of •OH was analyzed by TMB chromogenic reaction in pH, concentration, and time-dependent manners. The generation of ^1^O_2_ was determined by the DPBF chemical probe.

### 2.6 *In vitro* experiments

Cellular uptake of as-synthesized materials was operated on Panc02 cells. Cells were seeded in 6-well plates with a density of 1 × 10^5^ cells per well. The MTT cell assay was employed to evaluate the biocompatibility and toxicity of as-synthesized materials on L929 and Panc02 cells, respectively. Moreover, the live/dead cell assay was conducted to verify the cytotoxicity of the material on Panc02 cells. For intracellular ROS detection, a DCFH-DA chemical fluorescence probe was used. For the mitochondrial integrity assay, JC-1 staining kits were used to determine the J-monomer and J-aggregates separately. The intracellular fluorescence was observed by CLSM.

### 2.7 *In vivo* experiments

To investigate the biodistribution, the Panc02 tumor-bearing C57BL/6 mice were intravenously administered as-synthesized materials. For biodistribution investigation, the mice were sacrificed after 0, 2, 6, 12, 24, and 48 h. The heart, liver, lungs, spleen, kidneys, and tumors were collected for Cu contraction measurement. To estimate the anti-tumor efficacy of as-synthesized materials, the Panc02 tumor-bearing C57BL/6 mice were randomly placed into five groups (*n* = 5): control, CaO_2_, CaO_2_@Cu-ZIF, HA/CaO_2_-Ce6@Cu-ZIF, and HA/CaO_2_-Ce6@Cu-ZIF + Laser. During the treatment process, the tumor sizes and weights of mice were recorded once every 2 days: tumor volume = (tumor length) × (tumor width)^2^/2 (mm^3^).

### 2.8 Histology examination

After treatment process, the tumor and main organs (heart, liver, spleen, lung, and kidney) were collected for (H&E) staining according to the standard protocol for confirming caused injury.

### 2.9 Statistical analysis

All results were presented as mean ± S.D. Means were indicated using the student’s t-test. Statistical significance was determined at a value of *p < 0.05, **p < 0.01, ***p < 0.001.

## 3 Results and discussion

### 3.1 Characterization of HA/CaO_2_-Ce6@Cu-ZIF nanoplatform

**FIGURE 1:**
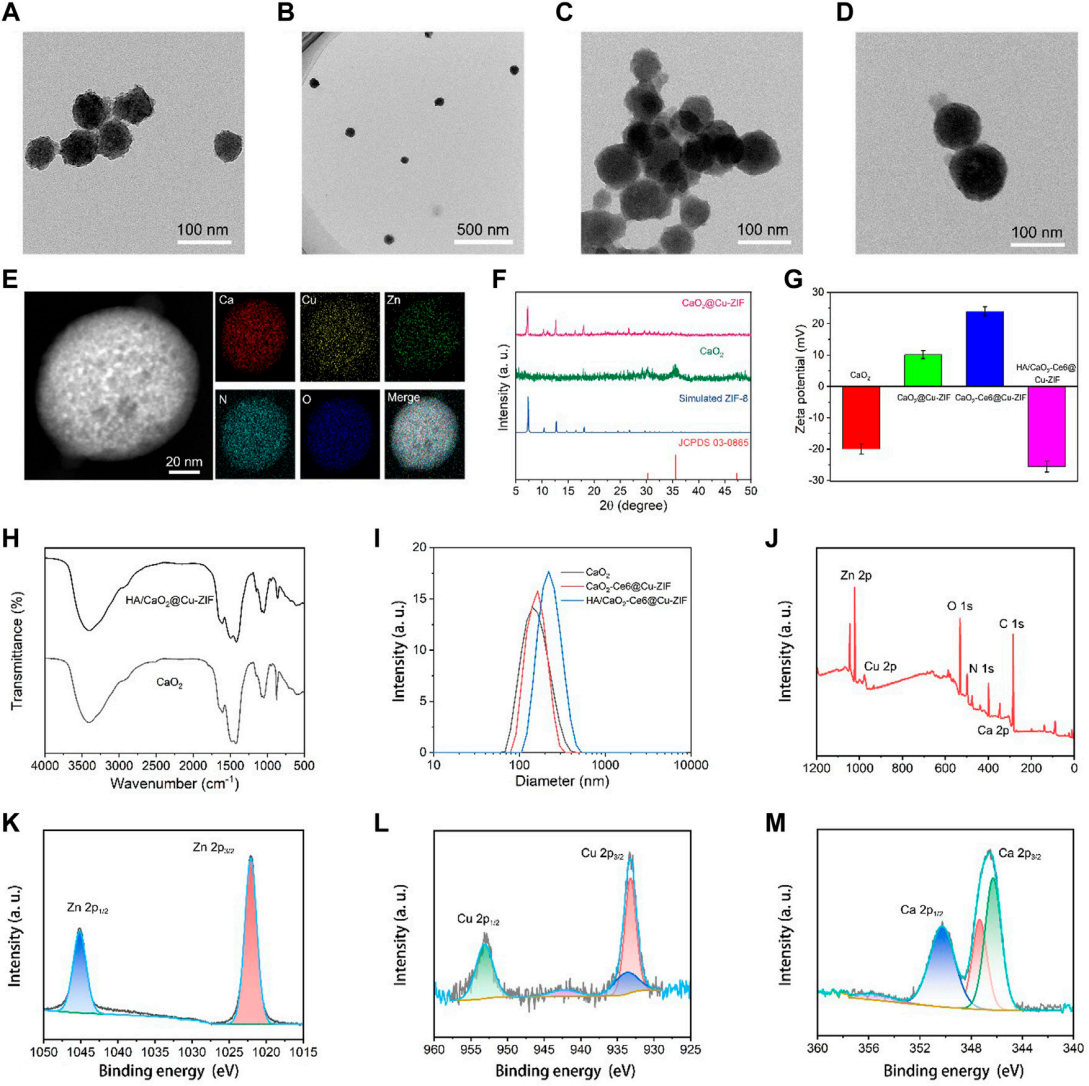
TEM images of **(A,B)** CaO_2_ NPs and **(C,D)** CaO_2_@Cu-ZIF. **(E)** Corresponding area-elemental mapping of CaO_2_@Cu-ZIF. **(F)** XRD patterns of stimulated ZIF-8, CaO_2_, and CaO_2_@Cu-ZIF. **(G)** Zeta potentials of CaO_2_, CaO_2_@Cu-ZIF, CaO_2_-Ce6@Cu-ZIF, and HA/CaO_2_-Ce6@Cu-ZIF. **(H)** FT-IR spectrum of CaO_2_ and HA/CaO_2_-Ce6@Cu-ZIF. **(I)** DLS of CaO_2_, CaO_2_-Ce6@Cu-ZIF, and HA/CaO_2_-Ce6@Cu-ZIF. **(J)** XPS spectrum of HA/CaO_2_-Ce6@Cu-ZIF. **(K–M)** High-resolution XPS spectrum of Zn, Cu, and Ca, respectively.

The synthesis of HA/CaO_2_-Ce6@Cu-ZIF was done through a two-step process. At first, CaO_2_ NPs were synthesized through a hydrolysis-precipitation process. Then, the HA/CaO_2_-Ce6@Cu-ZIF was synthesized through a simple one-step method. In detail, a specific Zn(NO_3_)_2_•6H_2_O and Cu(NO_3_)_2_•3H_2_O were dissolved in methanol and formed a uniform solution. Following this, 2-MeIM, HA-stabilized CaO_2_ NPs, and Ce6 dissolved in the methanol solution were added drop by drop and reacted for 30 min to obtain HA/CaO_2_-Ce6@Cu-ZIF. As revealed by transmission electron microscopy (TEM), the CaO_2_ NPs are about 90 ± 2.3 nm, demonstrating the uniform size distribution. (Figures 1A, B). X-ray diffraction (XRD) pattern reveals that the synthesized CaO_2_ NPs show obvious peaks at 30.1°, 35.6°, and 47.3° (Figure 1F), which is consistent with the JCPDS, No. 03-0865 according to previous literature for CaO_2_. (Sun et al., 2021a). After this, Cu-ZIF was utilized to encapsulate the CaO_2_ NPs and Ce6 via a self-assembly method to obtain the HA/CaO_2_-Ce6@Cu-ZIF nanoplatform. The TEM image shows that the HA/CaO_2_-Ce6@Cu-ZIF presents a regular octahedral shape with a particle size of around 110 ± 3.8 nm (Figures 1C, D). The homogeneous distributions of Zn, Cu, Ca, N, and O elements in HA/CaO_2_-Ce6@Cu-ZIF are revealed by the elemental mapping, which demonstrates the successful loading of CaO_2_ NPs (Figure 1E). Moreover, the XRD pattern of HA/CaO_2_-Ce6@Cu-ZIF is consistent with that of ZIF-8, indicating that the as-synthesized materials are well held in the crystal structure of ZIF-8 (Figure 1F). (Li et al., 2021b) To endow the CaO_2_-Ce6@Cu-ZIF with higher hydrophilicity for further biological application, HA with superior biocompatibility and targeted ability was employed for surface modification. As displayed in Figure 1G, the zeta potentials of CaO_2_, CaO_2_@Cu-ZIF, CaO_2_-Ce6@Cu-ZIF, and HA/CaO_2_-Ce6@Cu-ZIF are −20.03, +10.12, +23.9, and −25.6 mV, respectively, indicating that the CaO_2_ NPs and Ce6 are successfully introduced into the Cu-ZIF and HA are effectively modified on the surface of as-synthesized materials. Meanwhile, the size distribution of CaO_2_, CaO_2_-Ce6@Cu-ZIF, and HA/CaO_2_-Ce6@Cu-ZIF was obtained from the dynamic light scattering (DLS) measurements, the polydispersity index of which was 0.18, 0.19, and 0.17, respectively, demonstrating the good stable ability of HA modification. Figure 1I shows the hydrodynamic diameter is 142, 164, and 220 nm, respectively. The Fourier transform infrared (FT-IR) spectrum was recorded in the wavelength range of 500–4,000 cm^–1^ (Figure 1H), also suggesting the sequential addition of CaO_2_, Ce6, and HA, finally forming HA/CaO_2_-Ce6@Cu-ZIF. (Li et al., 2021a). As shown in X-ray photoelectron spectroscopy (XPS), HA/CaO_2_-Ce6@Cu-ZIF was also performed to evaluate the valence electron distribution, and the spectra are presented in which the coexistence of Zn, Cu, Ca, N, and O signals appear (Figure 1J). The high-resolution XPS of Zn, Cu, and Ca are shown in Figures 1K–M. In the high-resolution XPS of Cu spectrum, 933.3 and 953.6 eV peaks are assigned to Cu 2P3/2 and Cu 2p1/2, respectively. In addition, the satellite peaks at around 943.1 eV demonstrate the presence of Cu^2+^. (Li et al., 2021b). All the above materials’ characterizations imply the rational design and synthesis of H_2_O_2_/O_2_ self-supply and Ca^2+^ overloading MOF-based nanoplatform.

### 3.2 CDT/PDT synergistic effect of HA/CaO_2_-Ce6@Cu-ZIF nano platform

**FIGURE 2:**
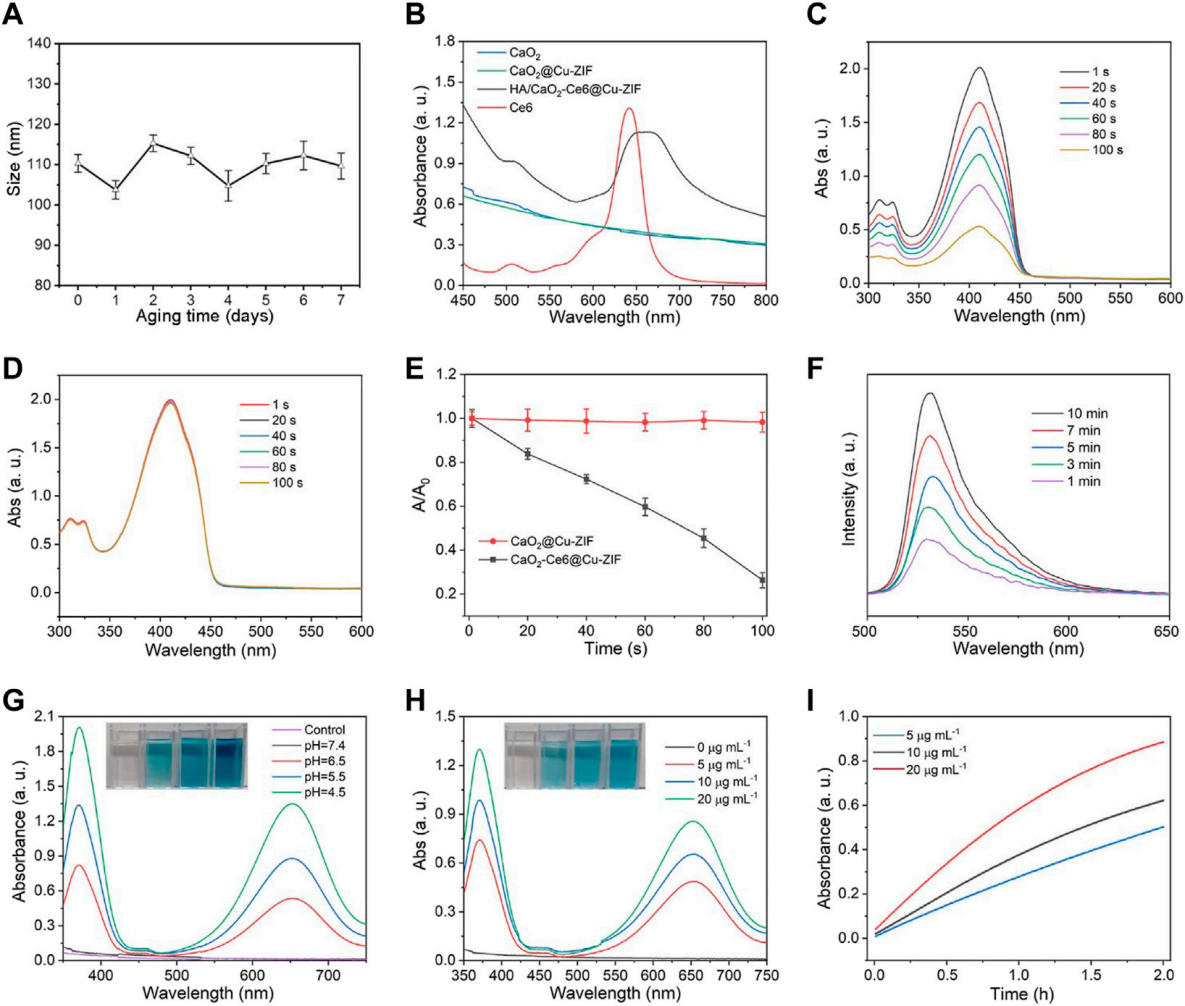
**(A)** Particle sizes of HA/CaO_2_-Ce6@Cu-ZIF within 7 days in cell medium. **(B)** UV-vis absorption spectra of Ce6, CaO_2_, CaO_2_@Cu-ZIF, and HA/CaO_2_-Ce6@Cu-ZIF. **(C)** UV-vis absorption of DPBF mixed with HA/CaO_2_-Ce6@Cu-ZIF as a function of reaction time under 650 nm laser irradiation. **(D)** UV-vis absorption of DPBF mixed with PBS at same condition. **(E)** Relative intensity value of UV-vis absorption peak at 410 nm for DPBF mixed with HA/CaO_2_-Ce6@Cu-ZIF and PBS, respectively. **(F)** Fluorescence spectra of DCFH-DA mixed with HA/CaO_2_-Ce6@Cu-ZIF under 650 nm laser irradiation for different time. **(G)** UV-vis spectra of TMB + HA/CaO_2_-Ce6@Cu-ZIF under pH at 4.5, 5.5, 6.5, and 7.4. **(H)** UV-vis spectra of TMB + HA/CaO_2_-Ce6@Cu-ZIF at the concentration of HA/CaO_2_-Ce6@Cu-ZIF as 0, 5, 15, and 20 μg/mL under pH 6.5. **(I)** UV-vis absorption peak at 650 nm for TMB + HA/CaO_2_-Ce6@Cu-ZIF at the concentration of HA/CaO_2_-Ce6@Cu-ZIF as 5, 15, and 20 μg/mL with different times. All laser pump powers are 0.5 W/cm^2^.

The stability experiments of HA/CaO_2_-Ce6@Cu-ZIF show that the as-synthesized materials maintain good dispersion within 7 days in cell medium (Figure 2A).The ultraviolet-visible (UV-vis) absorption spectra of Ce6, CaO_2_, CaO_2_@Cu-ZIF, and HA/CaO_2_-Ce6@Cu-ZIF was shown in Figure 2B. Compared with the broad peak of CaO_2_ and CaO_2_@Cu-ZIF ranging from 450 to 800 nm, the absorption band of HA/CaO_2_-Ce6@Cu-ZIF not only has the broad peak of CaO_2_@Cu-ZIF but also exhibits the typical characteristic peak of Ce6 around 650 nm. Encouraged by the results from the photo-properties of HA/CaO_2_-Ce6@Cu-ZIF, the ^1^O_2_ generation of PDT effect was explored by the UV-vis spectrum, where the 1,3-diphenylisobenzofuran (DPBF) was used as a real-time probe. The HA/CaO_2_-Ce6@Cu-ZIF and PBS solutions were irradiated by 650 nm laser (0.5 W/cm^2^), respectively. At first, the HA/CaO_2_-Ce6@Cu-ZIF could release Ce6 under acidic conditions. Then, DPBF could be oxidized by ^1^O_2_ which was generated from the combination of the released Ce6, light, and self-supplying O_2_, so that the absorption peak of the DPBF (the specific absorption wavelength was at 410 nm) gradually decreased along with time increase (Figure 2C). However, the absorption peak of the DPBF solution that was treated with PBS was almost unchanged (Figure 2D). The relative intensity value of the UV-vis absorption peak at 410 nm for DPBF mixed with HA/CaO_2_-Ce6@Cu-ZIF and PBS, respectively, further demonstrates the apparent decrease of DPBF absorption intensity (Figure 2E). To further confirm the production of ^1^O_2_, the 2′,7′-dichlorodihydrofluorescein diacetate (DCFH-DA) was also used (Figure 2F). And the results are consistent with the above. For •OH detection, a typical colorimetric analysis based on 3,3′,5,5′-tetramethyl-benzidine (TMB) was utilized to investigate the CDT effect of HA/CaO_2_-Ce6@Cu-ZIF. HA/CaO_2_-Ce6@Cu-ZIF can catalyze the oxidation of TMB to yield blue-colored oxTMB with typical absorbances at 370 and 652 nm. Considering the biodegradable properties related to the pH value of HA/CaO_2_-Ce6@Cu-ZIF, the influence of the pH on •OH generation was first analyzed (pH = 4.5, 5.5, 6.5, and 7.4). The result shows that the pH has a significant influence on the •OH generation (Figure 2G). There is no evident •OH generation at pH 7.4, while the ability of •OH generation remarkably increases with the downregulation of pH. Then the concentration effect of HA/CaO_2_-Ce6@Cu-ZIF for •OH generation was also investigated (Figure 2H). It shows an advanced ability of •OH generation along with the increased concentration (5, 15, and 20 μg/mL under pH 6.5). The •OH generation ability of HA/CaO_2_-Ce6@Cu-ZIF related to time was also investigated (Figure 2I).

### 3.3 *Invitro* experiments of HA/CaO_2_-Ce6@Cu-ZIF nanoplatform

**FIGURE 3:**
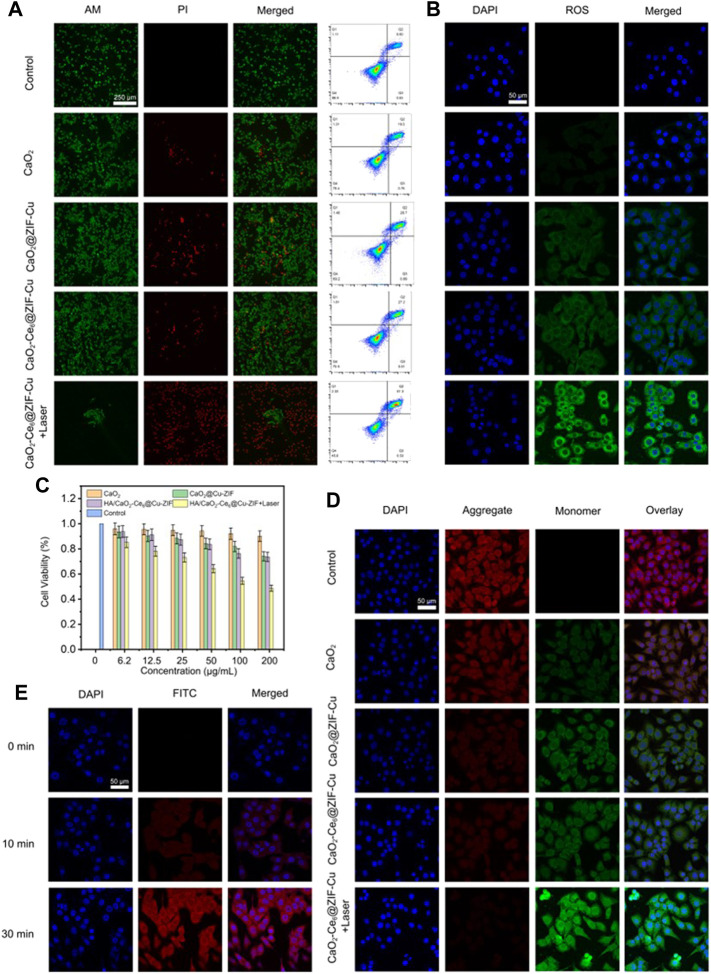
**(A)** Calcein-AM/PI double staining of Panc02 cells with different treatments and corresponding flow cytometry analysis by annexin V-FITC apoptosis detection kit. **(B)** Intracellular ROS level of Panc02 cells with different treatments. **(C)** Relative cell viabilities of Panc02 cells after treatment with different samples. **(D)** JC-1 staining of Panc02 cells after different treatments. **(E)** CLSM images of Panc02 cells incubated with HA/CaO_2_-Ce6@Cu-ZIF for different times.

Given the successful construction of HA/CaO_2_-Ce6@Cu-ZIF and advanced ROS generation capacity, the therapeutic effect of HA/CaO_2_-Ce6@Cu-ZIF against Panc02 cells *in vitro* was further investigated. The therapeutic performance was first examined through the calcein-AM and propidium iodide (PI) double-staining assay (Figure 3A). The confocal laser scanning microscopy (CLSM) images show that the HA/CaO_2_-Ce6@Cu-ZIF + Laser group exhibits the highest red-green ratio, where the red represents dead cells and green represents living cells, indicating the excellent anti-cancer effect of HA/CaO_2_-Ce6@Cu-ZIF. Meanwhile, the flow cytometric apoptosis assay with Annexin V-FITC and PI staining was used to calculate the apoptotic cell death mediated by HA/CaO_2_-Ce6@Cu-ZIF. The apoptotic ratio induced by HA/CaO_2_-Ce6@Cu-ZIF under irradiation was 51.83% (the sum of Q2 and Q3), which was markedly higher than other groups under the same condition. This is mainly attributed to synergistic H_2_O_2_/O_2_ self-supplying CDT/PDT synergistic effect. The intracellular ROS triggered by HA/CaO_2_-Ce6@Cu-ZIF under laser irradiation was further investigated using a 2,7-dichlorofluorescein diacetate (DCFH-DA) probe, which can be hydrolyzed to DCFH. This can be rapidly oxidized by the generated ROS and form DCF with green-fluorescent (excited by 488 nm). The CLSM images exhibit that there is almost no green fluorescence in the control and CaO_2_ groups. On the contrary, weak green fluorescence is exhibited in CaO_2_@Cu-ZIF and HA/CaO_2_-Ce6@Cu-ZIF groups. The strongest green fluorescence in the HA/CaO_2_-Ce6@Cu-ZIF + Lase group indicates that HA/CaO_2_-Ce6@Cu-ZIF under laser irradiation could generate more toxic ROS to induce tumor cell death (Figure 3B). The cytocompatibility of HA/CaO_2_-Ce6@Cu-ZIF on L929 normal cells was evaluated by the 3-(4,5-dimethylthiazol-2-yl)-2,5-diphenyltetrazolium bromide (MTT) method (tetramethylazole salt microenzyme reaction colorimetric assay). As shown in Supplementary Figure S1, HA/CaO_2_-Ce6@Cu-ZIF does not exhibit significant cytotoxicity to L929 cells, and the viability of cells treated with as-synthesized material for 24 h was 92.5% even at a concentration of 500 μg/mL, demonstrating the “silent” HA in the normal cellular microenvironment. Afterward, MTT assay was also used to estimate the cytotoxicity on Panc02 cells. Compared with others, the inhibition rate of HA/CaO_2_-Ce6@Cu-ZIF under laser irradiation is as high as 53.5%, where the concentration of HA/CaO_2_-Ce6@Cu-ZIF is 200 μg/mL (Figure 3C). Given that the Ca^2+^ overloading originating from CaO_2_ could further enhance the oxidative stress and result in mitochondrial dysfunction, the mitochondrial integrities of different treatment groups were examined through JC-1 staining flow cytometry (Figure 3D). The qualitative comparison of J-monomer (green) and J-aggregates (red) following various treatments shows that the group treated with HA/CaO_2_-Ce6@Cu-ZIF under laser irradiation exhibits abundant mitochondria damage. The endocytosis process of HA/CaO_2_-Ce6@Cu-ZIF in Panc02 cells was evaluated using specific fluorescence properties of Ce6. As is known, when excited with 488 nm light, the loaded Ce6 can radiate green fluorescence. As shown in Figure 3E, the results suggest that HA/CaO_2_-Ce6@Cu-ZIF could be effectively endocytosed by Panc02 cells and the internalization amount increased with prolonged time.

### 3.4 *In vivo experiments* of HA/CaO_2_-Ce6@Cu-ZIF nanoplatform

**FIGURE 4:**
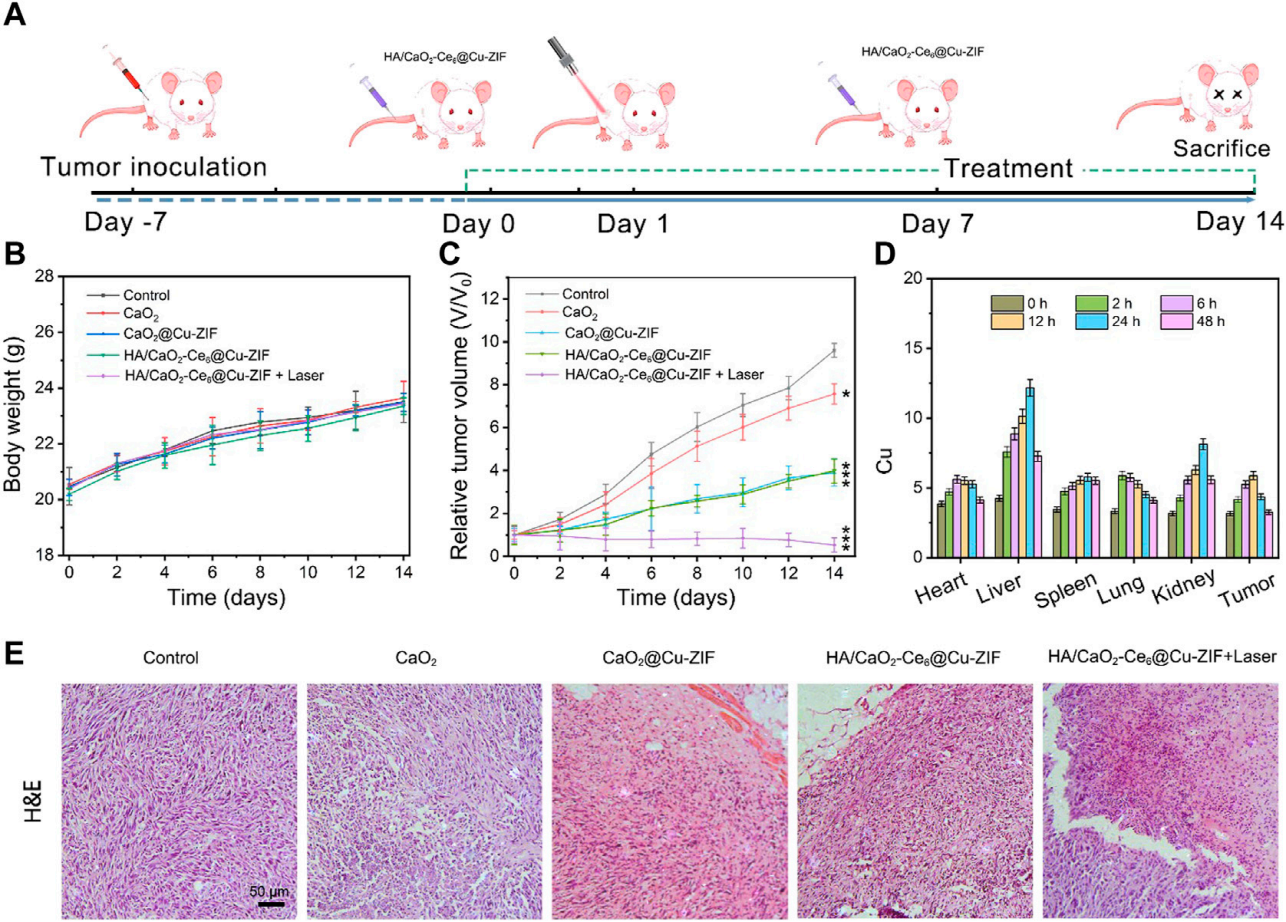
**(A)** Schematic illustration of Panc02 tumor-bearing mice model and treatment process. **(B)** Body weights and **(C)** relative tumor volume change curves of Panc02 tumor-bearing mice after various treatments. **(D)** Biodistribution of HA/CaO_2_-Ce6@Cu-ZIF in main organs and tumors at different time points (*n* = 3). **(E)** H&E-stained photographs of tumor slices obtained from Panc02 tumor-bearing mice in different groups after treatment.

**FIGURE 5:**
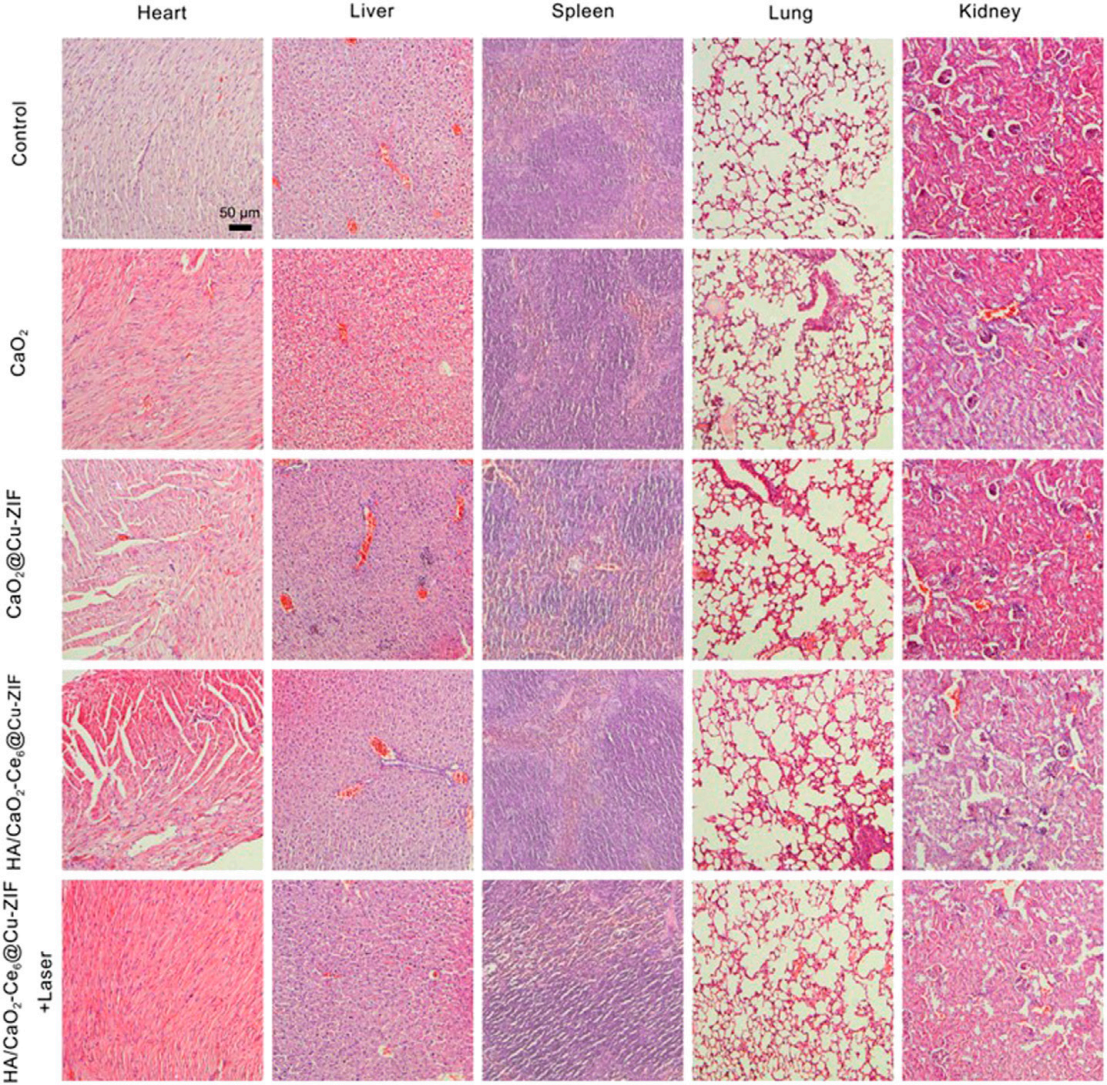
Representative H&E tissue sections from mice to monitor the histological change in heart, liver, spleen, lung, and kidney excised from different groups after treatment.

Inspired by the promising *in vitro* CDT/PDT synergistic effect of HA/CaO_2_-Ce6@Cu-ZIF nanoplatform, the *in vivo* therapeutic assay in Panc02 tumor-bearing C57BL/6 mice model was conducted. When the tumor sizes reached about 100 mm^3^, twenty-five Panc02 tumor-bearing mice were randomly divided into five groups, followed by treatments: control, CaO2, CaO_2_@Cu-ZIF, HA/CaO_2_-Ce6@Cu-ZIF, and v) HA/CaO_2_-Ce6@Cu-ZIF + Laser. As depicted in Figure 4A, Panc02 tumor-bearing mice were treated by intravenous administration on 1 and 7 days with injection doses of 15 mg/kg of mouse body weight. The body weight (Figure 4B) and tumor volume (Figure 4C) of mice were measured every 2 days during the treatment process. Moreover, the time-dependent Cu biodistribution of HA/CaO_2_-Ce6@Cu-ZIF at the tumor and major organs were evaluated (Figure 4D). The results indicate an effective accumulation of HA/CaO_2_-Ce6@Cu-ZIF at the tumor site, ensuring the following synergistic CDT/PDT therapeutics. In Figure 4B, during the treatment period, all the mice feature slight weight increases, demonstrating the negligible negative impacts of these treatments on the health of mice. As exhibited in Figure 4C, the relative tumor volume was notably suppressed in HA/CaO_2_-Ce6@Cu-ZIF + Laser group in comparison with the other groups. Specifically, the suppression rate of the HA/CaO_2_-Ce6@Cu-ZIF + Laser group was determined to be 60.8%, calculated from the variation in the relative tumor volume. This high suppression is attributed to the HA/CaO_2_-Ce6@Cu-ZIF induced cascade-amplified CDT/PDT therapy as follows: 1) CaO_2_ decomposed to generate H_2_O_2_ and O_2_, which alleviated the insufficiency of intracellular H_2_O_2_ and relieved hypoxia conditions in TME; 2) H_2_O_2_/O_2_ self-supplying effectively enhanced the production of •OH and ^1^O_2_ in Cu^2+^-mediated CDT and Ce6-induced PDT, respectively and 3) Ca^2+^ originated from CaO_2_ could further enhance the oxidative stress and result in mitochondrial dysfunction induced by Ca^2+^ overloading. Intensive therapeutic efficacy was also confirmed by hematoxylin and eosin (H&E) staining of tumor sections from each group (Figure 4E). The results were consistent with the above tumor growth data. Additionally, the histological observations of major organs (heart, liver, spleen, lung, and kidney) present negligible acute pathological toxicities and adverse effects during the treatment duration for the control or treated groups (Figure 5). These results demonstrate that HA/CaO_2_-Ce6@Cu-ZIF is of high biocompatibility.

## 4 Conclusion

In summary, a biodegradable HA/CaO_2_-Ce6@Cu-ZIF nanoplatform was rationally constructed for a H_2_O_2_/O_2_ self-supplying and Ca^2+^ overloading CDT/PDT synergistic strategy. After arriving at tumor sites via the specific HA targeted effect, HA/CaO_2_-Ce6@Cu-ZIF responded to acidic conditions in TME and released CaO_2_ NPs and Ce6, as well as exposed Cu^2+^ active sites within Cu-ZIF. The released CaO_2_ NPs further decomposed to efficiently generate H_2_O_2_ and O_2_ simultaneously for enhancing •OH and ^1^O_2_ production in Cu^2+^-mediated CDT and Ce6-participated PDT, respectively. In addition, the accompanying Ca^2+^ overloading generated by the decomposition of CaO_2_ NPs could induce mitochondrial dysfunction in tumor cells, further contributing to the combined CDT/PDT. Thus, this work provides an alternative strategy for smart reprogramming TME to improve the efficacy of synergistic CDT/PDT treatment.

## Data Availability

The original contributions presented in the study are included in the article/Supplementary Material, further inquiries can be directed to the corresponding authors.

## Supplementary material

SUPPLEMENTARY FIGURE S1 Biocompatibility of CaO2 @Cu-ZIF and HA/CaO2-Ce6@Cu-ZIF.
