# Supplementary figures and images for: H2O2/O2 self-supply and Ca2+ overloading MOF-based nanoplatform for cascade-amplified chemodynamic and photodynamic therapy
Source: Front Bioeng Biotechnol. 2023 May 24;11:1196839. doi: 10.3389/fbioe.2023.1196839 (PMC10245387; doi:10.3389/fbioe.2023.1196839)

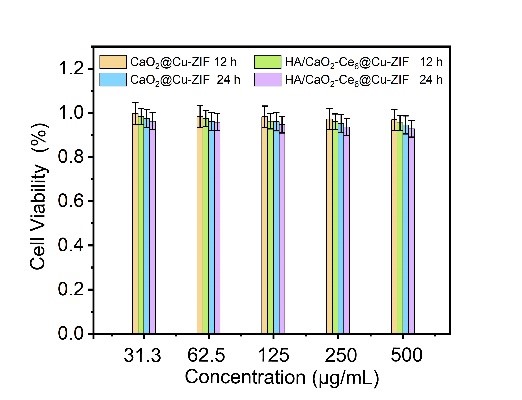

Supplement: Supplementary file 1 [file Image1.JPEG]
